# The interplay between vitamin D status and exerkine signaling: implications for exercise adaptation in athletes: narrative review

**DOI:** 10.1080/15502783.2026.2677647

**Published:** 2026-05-23

**Authors:** Do-Houn Kim

**Affiliations:** a Department of Human Physiology, Gonzaga University, Spokane, WA, USA

**Keywords:** Vitamin D, exerkines, myokines, vitamin D receptor (VDR), exercise adaptation

## Abstract

**Background:**

Exercise elicits systemic adaptations through a coordinated network of exercise-responsive signaling molecules termed exerkines. Vitamin D, classically linked to calcium homeostasis, has been increasingly characterized as a pleiotropic hormone with immunomodulatory and myotropic actions that may be relevant to training adaptation and recovery in athletic populations.

**Objective:**

This narrative review synthesizes mechanistic and clinical evidence examining whether vitamin D status and supplementation are associated with modulation of selected exercise-responsive exerkines, and it introduces a unifying conceptual model, the “vitamin D–exerkine axis”, to frame potential points of interaction between vitamin D signaling and the exercise-induced secretome.

**Methods:**

A narrative literature review was conducted using searches of PubMed and Google Scholar, incorporating mechanistic, observational, and intervention studies in animal and human models relevant to vitamin D signaling and exercise-responsive exerkines. Evidence was synthesized qualitatively to distinguish biological plausibility from athlete-specific causal inference.

**Results:**

Across experimental systems, vitamin D signaling via the vitamin D receptor (VDR) has been associated with expression of several exerkines implicated in inflammation, metabolism, and muscle remodeling. Evidence most consistently discussed in the literature involves IL-6, irisin/FNDC5, myostatin, and anti-inflammatory cytokines (e.g. IL-10), although effect direction and magnitude appear context-dependent and are influenced by baseline vitamin D status, study design, and outcome timing. A bidirectional relationship is plausible: exercise may upregulate VDR expression in skeletal muscle and has been associated with transient changes in circulating vitamin D metabolites, while vitamin D sufficiency may shape aspects of the post-exercise inflammatory and metabolic milieu. Collectively, these observations support a working model in which vitamin D status could modulate parts of the exercise-response signaling network, but definitive athlete-focused causal evidence remains limited.

**Conclusion:**

The proposed vitamin D–exerkine axis offers a hypothesis-generating conceptual model for integrating nutrition endocrinology with exercise physiology. Current data support biological plausibility for interaction, yet heterogeneity in study populations, endpoints, and supplementation protocols constrains strong causal inference in athletes. Future research should prioritize well-controlled trials that account for baseline 25(OH)D status, define dose–response relationships, test sex- and sport-specific effects, and incorporate tissue-level endpoints to clarify mechanisms and relevance to training adaptation and recovery.

## Graphical abstract

**Figure:**
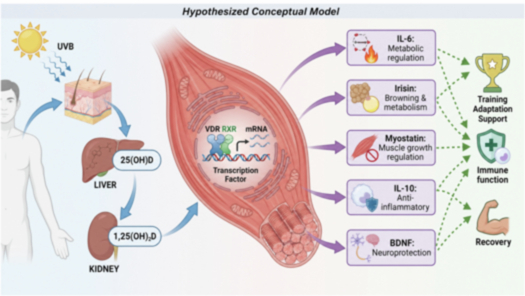

The Proposed Vitamin D-Exerkine Axis Pathway. This schematic outlines a hypothesis-generating conceptual model by which vitamin D may interact with exercise-responsive exerkines. Solar UVB exposure initiates the synthesis of vitamin D, which is activated in the liver and kidneys to form 1,25(OH)2D. In skeletal muscle, 1,25(OH)2D binds to the VDR, which acts as a transcription factor to modulate the expression of key exerkines, including IL-6, Irisin, Myostatin, IL-10, and BDNF. While this theoretical modulation of the exerkine profile offers a biologically plausible framework for supporting immune function and recovery, immune function, and recovery, direct causal evidence in trained athletes remains limited. **Abbreviations:** 25(OH)D, 25-hydroxyvitamin D; 1,25(OH)2D, 1,25-dihydroxyvitamin D; BDNF, Brain-Derived Neurotrophic Factor; IL-6, Interleukin-6; IL-10, Interleukin-10; VDR, Vitamin D Receptor.

## 1. Introduction

### 1.1. The exercise as medicine concept

Physical activity is a foundational element of human health. The contemporary view of “Exercise is Medicine” has established it as a cornerstone of preventive and therapeutic healthcare [1,2]. Substantial evidence shows that consistent physical activity improves health span and helps prevent many chronic diseases [3,4]. Lifelong physical activity is associated with a delayed onset of at least 40 chronic conditions, including heart disease, type 2 diabetes, and obesity, highlighting its significant public health impact [2,5]. The systemic benefits of physical activity, affecting nearly every organ system, are coordinated through inter-organ communication [6,7]. Contracting skeletal muscle sends signals to distant organs, triggering system-wide responses that enhance metabolic function, reduce inflammation, and improve recovery capacity. This brings up a key question: how are these widespread effects coordinated? The answer appears to lie in a refined biological signalling system involving molecular messengers.

### 1.2. Identifying the messengers: the growth of exerkine research

The search for an “exercise factor”, a humoral signal from muscle that madidates the metabolic effects of physical activity, has long been an objective in physiology [8]. This perspective shifted in 2000 with the discovery that contracting skeletal muscle produces and releases interleukin-6 (IL-6) into the circulation, establishing muscle as an endocrine organ [8,9]. This finding led to the classification of such signalling molecules as “myokines.” The term “exerkine” was later introduced in 2016 to provide a broader classification of signalling molecules released in response to physical activity, not only from muscle but from many tissues [10]. Exerkines are now recognised as a diverse class of molecules, including proteins, nucleic acids, and metabolites, released from skeletal muscle (myokines), adipose tissue (adipokines), liver (hepatokines), and other organs. These molecules mediate the systemic effects of physical activity through endocrine, paracrine, and autocrine signalling [10].

### 1.3. Vitamin D: the expanding functions of the sunshine hormone

At the same time as the growth of exerkine research, the scientific understanding of vitamin D has undergone a marked shift. Traditionally known for its essential role in calcium homoeostasis and bone metabolism, vitamin D is recognised as a hormone with pleiotropic effects beyond the skeleton [11,12]. The vitamin D receptor (VDR) is expressed in many tissues, including immune cells and skeletal muscle, indicating a broader physiological influence than previously recognised [13]. Growing evidence supports vitamin D's role as a modulator of immune function, inflammation, and muscle health [13,14]. This expanded role is particularly relevant to physical activity, as a high prevalence of vitamin D deficiency has been reported among athletes across various sports, with potential negative effects for performance and injury risk [15,16]. The convergence of these two rapidly advancing fields of study, exerkines and vitamin D, provides a strong reason for a more integrated investigation.

### 1.4. Justification and area of the review

It should be noted that the evidence base for the proposed vitamin D–exerkine axis is primarily mechanistic, derived from cell and animal models, with a smaller body of human data drawn largely from non-athlete clinical populations; therefore, proposed clinical applications should be interpreted with this limitation in mind.

While research on vitamin D and exerkines has progressed separately, their intersection remains largely unexplored. Evidence suggests a cooperative relationship, in which physical activity may enhance the protective effects of vitamin D [17], and sufficient vitamin D levels may, in turn, improve the body's response to physical activity by modulating inflammatory and metabolic signalling pathways [13,18]. This has led to the proposal of a “Vitamin D-Exerkine Axis”, a theoretical model describing the intersecting signalling pathways through which vitamin D and exercise-induced factors may jointly regulate health [19]. To date, no narrative review has comprehensively integrated the evidence supporting this axis. This review aims to address that gap. Specifically, it seeks to: 1) describe the key components of the Vitamin D-Exerkine axis; 2) synthesise mechanistic and clinical evidence on their interactions in relation to health, physical performance, and disease; and 3) provide an integrated perspective to guide future research and practical strategies for optimising health through the combined effects of vitamin D and physical activity.

## 2. Materials and methods

### 2.1. Study design and scope

This manuscript was prepared as a narrative review intended to (i) summarise current evidence connecting vitamin D status/supplementation with exercise-responsive signalling molecules (exerkines) and (ii) develop a conceptual framework for the proposed “vitamin D–exerkine axis,” rather than to exhaustively identify all eligible studies or formally rate study quality, as would be expected in a systematic review. The approach was designed to integrate mechanistic biology with athlete-relevant applied considerations in sports nutrition.

### 2.2. Information sources and search strategy

A literature search was conducted using the electronic databases PubMed and Google Scholar, consistent with the review’s stated aim to capture both biomedical and broader interdisciplinary sports science literature. Source Searches were performed from database inception through 31 December 2025, with additional targeted searching performed during manuscript preparation to identify foundational (“seminal”) papers and recent reviews relevant to vitamin D signalling and the exercise-induced secretome.

Key terms were applied in varying combinations and, where appropriate, using Boolean operators and quotation marks. Search concepts included: “vitamin D”, “25-hydroxyvitamin D” or “25(OH)D”, “1,25-dihydroxyvitamin D” or “1,25(OH)2D”, “vitamin D receptor” or “VDR”, “exercise”, “training”, “athlete”, “exerkine”, “myokine”, “adipokine”, “hepatokine”, “osteokine”, and exerkine candidates emphasised in this review, including interleukin-6 (IL-6), irisin/FNDC5, myostatin (GDF-8), brain-derived neurotrophic factor (BDNF), interleukin-15 (IL-15), interleukin-10 (IL-10), adiponectin, leptin, resistin, fibroblast growth factor 21 (FGF21), osteocalcin, FGF23, and Klotho.

For clarity, throughout this review, ‘vitamin D’ refers collectively to the vitamin D system, including vitamin D₂ (ergocalciferol) and vitamin D₃ (cholecalciferol); ‘25(OH)D’ (calcidiol) denotes the primary circulating form used to assess vitamin D status; and ‘1,25(OH)₂D’ (calcitriol) denotes the biologically active form that binds the VDR. Where studies report specific metabolites, the precise form is specified.

To enhance topic coverage in a narrative framework, reference lists of highly relevant review articles and key primary studies were also screened to identify additional publications not captured by initial keyword combinations (citation chaining). Priority was given to literature that directly informed the mechanistic plausibility and athlete-facing interpretation of the proposed axis.

Given the rapidly evolving nature of exerkine research, priority was given to studies published from 2010 onward. Earlier publications were included only when they provided foundational mechanistic context, particularly regarding VDR signalling and cytokine regulation, that has not been superseded by more recent work.

### 2.3. Eligibility and selection

Because the objective was conceptual synthesis rather than systematic enumeration, study selection was based on relevance to the vitamin D–exerkine axis and sports nutrition translation. I included peer-reviewed publications that met one or more of the following criteria: (1) mechanistic studies (in vitro or animal) describing how vitamin D metabolites and/or VDR signalling influence expression, secretion, or downstream signalling of exercise-responsive molecules; (2) observational studies associating vitamin D status (typically circulating 25(OH)D) with exerkine-related outcomes or exercise-responsive inflammatory/metabolic markers; and (3) intervention studies examining vitamin D supplementation in the context of exercise, training adaptation, recovery, inflammation, immune outcomes, or exerkine responses. Both human and animal model evidence was considered to support mechanistic interpretation, with athlete-focused and exercise-intervention human studies emphasised when available.

I excluded non-peer-reviewed sources where the underlying data could not be evaluated (e.g. non-scholarly web content), and studies that did not provide a clear link between vitamin D status/supplementation (or VDR signalling) and exercise-responsive signalling pathways or candidate exerkines. Articles focused exclusively on vitamin D and bone outcomes without mechanistic or applied relevance to exercise-responsive signalling were not prioritised unless they informed athlete-relevant clinical context (e.g. stress fracture risk). Given the narrative design, no PRISMA flow diagram was produced, and no formal risk-of-bias tool was applied; instead, divergent findings were addressed qualitatively by considering study design features such as baseline vitamin D status, dosing regimen, population/training status, and timing of outcome assessment relative to exercise.

### 2.4. Data handling and synthesis approach

Information was synthesised qualitatively and organised thematically to support the conceptual framework of the vitamin D–exerkine axis. Specifically, evidence was integrated across (i) foundational definitions and classification of exerkines, (ii) vitamin D metabolism and VDR signalling relevant to muscle and immune function, (iii) proposed bidirectional interactions between exercise and vitamin D signalling, and (iv) candidate exerkines highlighted in this review (e.g. IL-6, irisin, myostatin, IL-10 and related mediators). Source Emphasis was placed on clearly distinguishing biological plausibility derived from mechanistic studies from athlete-specific causal inference, which remains limited and represents a key area for future controlled trials.

## 3. The world of exerkines: a foundational overview

### 3.1. Defining and classifying the exerkinome

The term exerkine is defined as a “signalling moiety released in response to acute and/or chronic exercise that exerts their effects through endocrine, paracrine and/or autocrine pathways” [10]. This classification encompasses a wide range of molecules, including proteins, nucleic acids, and metabolites, secreted by multiple tissues during and after physical activity [10,20]. The complete set of these exercise-induced factors is referred to as the exerkine.

Exerkines are categorised based on their tissue of origin, providing a framework to organise the complex network of inter-organ communication induced by physical activity. Table 1 summarise the major exerkine categories, with examples of specific molecules in each class, including their relationship with vitamin D.

**Table 1.** Classification of exerkines by tissue of origin and relationship with vitamin D.

Category	Tissue of origin	Example exerkines	Vitamin D relationship (evidence summary)
Myokines	Skeletal muscle	Interleukin-6 (IL-6), irisin (FNDC5-derived), myostatin (GDF-8), brain-derived neurotrophic factor (BDNF), interleukin-10 (IL-10), interleukin-15 (IL-15)	IL-6: VDR-mediated transcriptional regulation; effect on post-exercise response depends on baseline vitamin D status [27,37,58,61,66]. Irisin: Vitamin D increases irisin via Sirt1/PGC-1α pathway [29,67,68,69]. Myostatin: Inverse association; vitamin D supplementation reduces myostatin in animal models [30,71]. BDNF: Synergistic effects of vitamin D and exercise on BDNF expression in animal models [28,75]. IL-10: Vitamin D promotes IL-10 production via regulatory T-cell programming; higher post-exercise IL-10 in vitamin D-sufficient individuals [36,72,73,74].
Adipokines	Adipose tissue	Adiponectin, leptin, resistin	Adiponectin: Positive association with vitamin D status in metabolic populations; independent effects in lean athletes unclear [31,32,33]. Leptin: Variable relationship; vitamin D supplementation associated with reduced leptin in some obese populations; confounded by adiposity [31,32]. Resistin: Inverse association with vitamin D status reported in metabolic syndrome populations; athlete-specific data limited.
Hepatokines	Liver	Fibroblast growth factor 21 (FGF21)	FGF21 and vitamin D both regulate glucose and lipid metabolism; potential for crosstalk in metabolic adaptation, but direct mechanistic link in exercise context not well-defined. Evidence primarily from metabolic disease models rather than exercise physiology.
Osteokines	Bone	Osteocalcin, fibroblast growth factor 23 (FGF23), Klotho	FGF23-Klotho axis: Vitamin D regulates phosphate/vitamin D endocrine feedback loop via FGF23 and Klotho [35]. Osteocalcin: Vitamin D influences osteocalcin carboxylation and activity; osteocalcin implicated in energy metabolism and muscle function. Relevance to exercise-induced bone signalling and stress fracture risk in athletes requires investigation.
Other kines	Various tissues	Cardiokines (heart), neurokines (neurons), batokines (brown adipose tissue), inflammatory cytokines (immune cells)	Inflammatory cytokines (TNF-*α*, IL-1β): Vitamin D inhibits NF-κB pathway, reducing pro-inflammatory cytokine production [59,60,61]. VDR expressed in multiple tissues including heart, neurons, and immune cells, suggesting broad potential for vitamin D modulation of tissue-specific exercise-responsive signals. Mechanistic pathways in exercise context largely unexplored.

**Note:** This table provides a foundational classification of exerkines by tissue of origin. The vitamin D relationship column summarises evidence discussed in detail in subsequent sections and Table 2. References in brackets correspond to the manuscript reference list. Evidence quality and athlete-specificity vary by exerkine; see Table 2 for detailed evidence tier designations. **Abbreviations:** BDNF, brain-derived neurotrophic factor; FGF21, fibroblast growth factor 21; FGF23, fibroblast growth factor 23; FNDC5, fibronectin type III domain-containing protein 5; GDF-8, growth differentiation factor 8; IL, interleukin; NF-κB, nuclear factor kappa B; PGC-1α, peroxisome proliferator-activated receptor gamma coactivator 1-alpha; Sirt1, sirtuin 1; TNF-α, tumour necrosis factor alpha; VDR, vitamin D receptor.

### 3.2. Modes of action

Exerkines exert their effects through several signalling mechanisms that enable precise regulation of cellular and systemic responses. These mechanisms are:

- Endocrine signalling: Exerkines are released into the bloodstream and act on distant target cells [10].
- Paracrine signalling: Exerkines act on nearby cells by diffusing through the extracellular space [10].
- Autocrine signalling: Exerkines act on the same cell that released them, forming a self-regulatory feedback loop [10].

**Figure 1.**
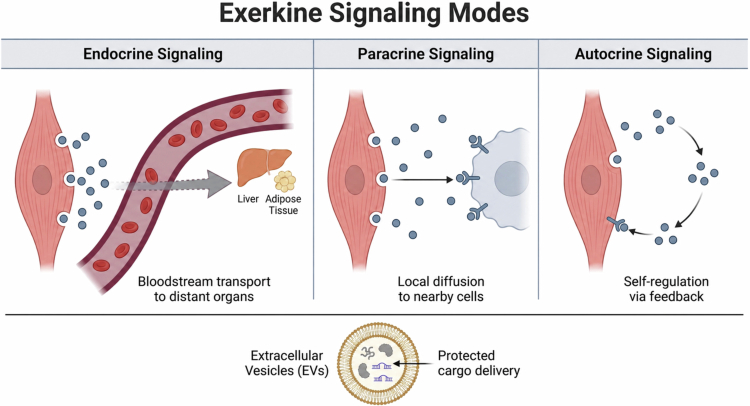
Exerkine signalling modes. This figure illustrates the three primary modes of exerkine signalling. (A) Endocrine: Exerkines are released from muscle cells into the bloodstream to act on distant organs like the liver and adipose tissue. (B) Paracrine: Exerkines diffuse locally to act on adjacent cells. (C) Autocrine: Exerkines bind to receptors on the same cell that released them, creating a self-regulatory feedback loop. Extracellular Vesicles (EVs) are shown as a mechanism for protected cargo delivery of exerkines and other signalling molecules.

Many exerkines are also transported within extracellular vesicles (EVs), small membrane-bound particles released from cells that carry proteins, lipids, and nucleic acids [21]. EVs functions as carriers of molecular signals, protecting their cargo from degradation and facilitating inter-organ communication following physical activity [21,22]. Studies indicate that skeletal muscle cells release EVs enriched with specific protein and microRNAs, suggesting a targeted mechanism for transmitting exercise-induced signals [23] (Figure 1).

### 3.3. Functional roles in athletic contexts

**Figure 2.**
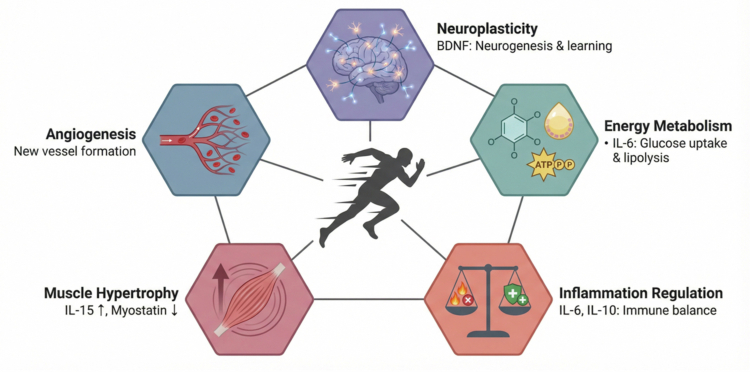
Functional roles of exerkines in athletes. This figure summarises five key functions of exerkines relevant to athletic contexts, all contributing to an integrated physiological response. These include the regulation of (1) Energy Metabolism, (2) Inflammation, (3) Muscle Hypertrophy, (4) Angiogenesis (new blood vessel formation), and (5) Neuroplasticity (neuronal growth and adaptation).

The release of exerkines during physical activity coordinates range of physiological adaptations relevant to athletic populations. These molecules are central to training adaptation and performance maintenance. Their functions include:

- Energy Metabolism: Exerkines regulate energy availability. For instance, the myokine IL-6 regulates lipolysis in adipose tissue and hepatic glucose production, ensuring a steady supply of fuel for working muscles [7]. Other exerkines enhance glucose uptake and fatty acid utilisation, contributing to metabolic regulation [20].
- Inflammation: Physical activity induces a complex inflammatory response, with exerkines acting as key modulators. Muscle-derived IL-6 exerts anti-inflammatory effects that counterbalance pro-inflammatory states with intense or prolonged activity [7]. This regulation is important for recovery and adaptation.
- Muscle Hypertrophy: Exerkines influence muscle growth and repair. Myokines such as IL-15 anabolic effects, while myostatin negatively regulator of muscle mass [24]. The balance of these signals, influenced by training, determines overall muscle size and strength.
- Angiogenesis: Exerkines contribute to the formation of new blood vessels, enhancing oxygen and nutrient delivery to muscle. Exerkines are involved in stimulating this process, improving endurance capacity and cardiovascular function [25].
- Neuroplasticity: Exerkines also mediate the effects of physical activity on brain health. BDNF, a myokine, supports neurogenesis and synaptic plasticity, processes essential for learning and memory [26] (Figure 2).

### 3.4. The vitamin D and exerkine connection

Recent research highlights a significant interplay between vitamin D and the exerkinome. Vitamin D, via its receptor (VDR) expressed in multiple tissues including skeletal muscle, modulate the production and function of various exerkines. This interaction adds another layer of complexity to the body's response to exercise.

- Myokine Modulation: Vitamin D status is associated with several key myokines. For instance, vitamin D modulates the inflammatory and metabolic actions of IL-6 [27]. It increase the expression of irisin and BDNF, which have beneficial effects on metabolism and neuroprotection [28,29]. Furthermore, vitamin D metabolites may influence the exercise-induced reduction in myostatin, a negative regulator of muscle growth [30].
- Adipokine Regulation: Vitamin D also is potentially linked to release of adipokines. Vitamin D supplementation has been associated with increased adiponectin, an adipokine with anti-inflammatory and insulin-sensitising properties [31]. Conversely, vitamin D levels are inversely associated with the pro-inflammatory adipokines leptin and resistin [32,33].
- Bone-Derived Factors: Vitamin D is involved in pathways related to osteokines. Vitamin D is essential for the function of osteocalcin, an osteokine which also acts as an exerkine influencing muscle function [34]. Moreover, vitamin D regulates the FGF23-Klotho axis, a bone-kidney endocrine system that governs phosphate and vitamin D metabolism [35].
- Inflammatory Mediators: Beyond specific cytokines, vitamin D is associated with broader effects on inflammatory mediators within the exerkinome. It reduces levels of pro-inflammatory cytokines such as TNF-*α* and IL-1β, which are also modulated by exercise [36,37].

This evidence suggests that adequate vitamin D status may optimise the body's response to exercise by fine-tuning the exerkinome. The following sections examine this “Vitamin D-Exerkine Axis” in greater detail.

As depicted in Figure 4, this modulation is proposed to occur through a multi-step pathway. Exercise increases VDR expression in skeletal muscle and immune cells. Circulating 1,25(OH)₂D then binds VDR, forming an activated receptor–ligand complex that may inhibit NF-κB translocation to the nucleus and reduce transcription of pro-inflammatory genes. This process may shift the post-exercise exerkine profile toward a more regulatory, anti-inflammatory state. Each step represents a distinct, testable mechanism that requires validation in human athletic populations.

## 4. Vitamin D: a pleiotropic regulator in sports nutrition

### 4.1. From sunlight to signaling: vitamin D metabolism and the VDR

Vitamin D synthesis is a multi-stage process that begins in the skin and culminates in the formation of a steroid hormone. The initial stage involves the conversion of 7-dehydrocholesterol to pre-vitamin D3 upon exposure to ultraviolet B (UVB) radiation, followed by temperature-dependent isomerization to vitamin D3 (cholecalciferol) [38]. This cutaneous production is non-enzymatic. Vitamin D3 is then transported to the liver for initial hydroxylation, where the enzyme CYP2R1 converts it into 25-hydroxyvitamin D [25(OH)D] [39,40], the main circulating form of vitamin D used to assess vitamin D status.

The final activation step occurs primarily in the kidneys, where 1α-hydroxylase (CYP27B1) converts 25(OH)D into the biologically active form, 1,25-dihydroxyvitamin D [1,25(OH)2D] [38]. This active form exerts its effects primarily via vitamin D Receptor (VDR), a member of the nuclear steroid-thyroid hormone receptor superfamily [41]. Upon binding, VDR forms a heterodimer with the retinoid X receptor (RXR), which binds to vitamin D response elements (VDREs) in target gene promoters to regulate transcription [41]. The VDR is expressed in a wide range of tissues, including skeletal muscle and immune cells such as T and B lymphocytes, macrophages, and dendritic cells, suggesting broad regulatory function beyond bone homoeostasis [42,43].

### 4.2. Extra-skeletal functions in athletes

The widespread distribution of the VDR enables 1,25(OH)2D to influence numerous physiological processes relevant to athletic performance, including muscle function and immune responses.

*Muscle function.*

Experimental evidence suggests a direct relationship between VDR activation and muscle regulation. Overexpression of VDR in mouse models has been shown to induce skeletal muscle hypertrophy, attributed to increased protein synthesis and ribosomal biogenesis [44]. Additionally, 1,25(OH)2D promotes myogenic differentiation of satellite cells, which are essential for muscle repair and regeneration [44].

Vitamin D status is associated with muscle fibre composition. Vitamin D deficiency is linked to atrophy of type II (fast-twitch) muscle fibers [45], where vitamin D3 supplementation is associated with increased muscle fibre size and nuclear VDR concentration in older adults with limited mobility [45]. Animal studies further support these findings; VDR knockout mice exhibit abnormal skeletal muscle development and altered expression of myogenic regulatory factors [46].

Importantly, vitamin D supplementation has well-established, independent clinical benefits in severely deficient adults, including improvements in muscle strength and physical performance. Meta-analysis suggests these effects are modest but meaningful [47]. These established effects should be considered alongside the proposed exerkine-mediated mechanisms which remain less certain. Practitioners should therefore distinguish these pathways when interpreting supplementation outcomes.

*Immune modulation.*

Vitamin D is recognised to exert significant effects on both the innate and adaptive immune systems. VDR is expressed in most immune cells, allowing 1,25(OH)2D to modulate immune function [43,48]. In innate immunity, 1,25(OH)2D increases the production of antimicrobial peptides such as cathelicidin in macrophages, strengthening the first line of defence against pathogens [49].

**Figure 3.**
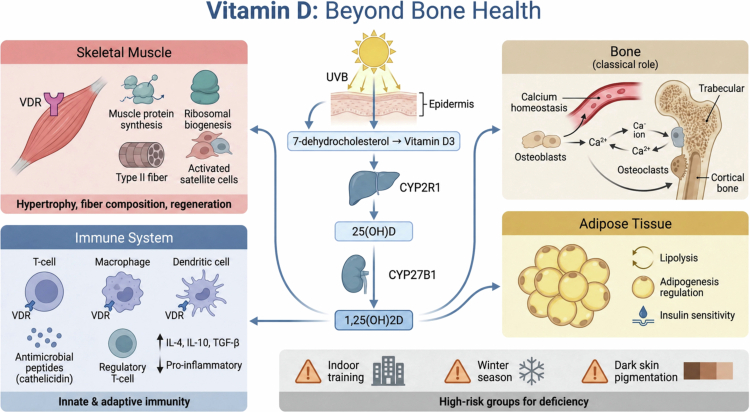
Vitamin D: a pleiotropic regulator. This figure depicts the synthesis pathway of active vitamin D (1,25(OH)2D) and its widespread, or pleiotropic, effects beyond classical bone health. It highlights the regulatory role of vitamin D in skeletal muscle, the immune system, and adipose tissue. At the bottom, it identifies key high-risk groups for vitamin D deficiency in athletes, including those with limited sun exposure due to indoor training or winter season, and individuals with darker skin pigmentation.

In adaptive immunity, 1,25(OH)2D modulates T-cell activation and the function of antigen-presenting cells like dendritic cells [48,50], promoting a shift towards a more anti-inflammatory state. This is partially mediated by increased production of anti-inflammatory cytokines (e.g. interleukin-4 [IL-4], interleukin-10 [IL-10], and transforming growth factor-beta [TGF-*β*]) and reduced expression of pro-inflammatory cytokines [36]. This balanced regulation of inflammation may be particularly relevant for athletes, who frequently experience exercise-induced inflammation (Figure 3).

### 4.3. The athlete's paradox: high physical demands, low vitamin D status

Despite the recognised functions of vitamin D in processes relevant to athletic performance, inadequate vitamin D status is common in athletes. Vitamin D insufficiency is typically defined as a serum 25(OH)D concentration below 30 ng/mL (75 nmol/L), while deficiency as below 20 ng/mL (50 nmol/L) [51]. Meta-analyses estimates that up to 56% of athletes have inadequate vitamin D levels [52].

Several factors contribute to this prevalence, creating a paradox in which individuals with high physical demands are often suboptimal in a key nutrient affecting physiological function. These include:

- Indoor Sports: Athletes training primarily indoors have limited UVB exposure, the main source of cutaneous vitamin D synthesis, and consistently show lower serum 25(OH)D levels than outdoor atheletes [53].
- Winter Training and Geographic Latitude: Seasonal variation and latitude heavily influence UVB availability. During winter and at higher latitudes, UVB radiation is insufficient for vitamin D synthesis, leading to a predictable decline in vitamin D status [53,54].
- Skin Pigmentation: Melanin reduces cutaneous vitamin D3 from UVB exposure. Individuals with darker skin require longer sun exposure to produce equivalent vitamin D levels, increasing the risk for insufficiency [54].

This widespread inadequacy may impair training capacity, recovery, and overall performance.

## 5. The vitamin D-exerkine axis: mechanistic crossroads

This section examines the molecular interactions between vitamin D signalling and exercise-induced factors. It is important to acknowledge the profound heterogeneity of these mediators. Molecules such as IL-6, irisin, and BDNF may differ in their tissue of origin, release kinetics, assay reliability and biological context. The proposed axis represents a theoretical intersection of vitamin D with multiple, distinct exercise-responsive pathways, rather than ta definitely established, unified endocrine architecture with shared regulatory rules.

### 5.1. The VDR: a transcriptional regulator in skeletal muscle

The Vitamin D Receptor (VDR) mediates the effects of vitamin D in skeletal muscle. Its expression is dynamically regulated by physical exercise, altering muscle responsiveness to circulating 1,25(OH)2D. Both acute and chronic exercise has been shown to increase the expression of VDR in muscle tissue. For example, a single bout of resistance exercise upregulated VDR protein expression in rat skeletal muscle within hour [55], while high-intensity interval exercise increased VDR expression, particularly in satellite cells involved in muscle repair and adaptation [56]. This exercise-induced upregulation may enhance muscle sensitivity to vitamin D, potentially amplifying downstream effects without change in circulating vitamin D levels.

Upon binding of 1,25(OH)2D, the VDR-ligand complex translocates to the nucleus and forms a heterodimer with the retinoid X receptor (RXR). This complex binds to vitamin D response elements (VDREs) in the promoter regions of target genes [44]. VDR regulates genes encoding exerkines; for example, exercise-induced VDR expression has been linked to the suppression of interleukin-6 (IL-6) mRNA in skeletal muscle, indicating a direct transcriptional link between VDR and this myokine [57]. This regulatory role positions VDR as a key mediator of the muscular response to exercise.

### 5.2. How vitamin D shapes the exercise-induced milieu

Physical exercise, particularly when strenuous, induces a transient inflammatory state characterised by the release of signalling molecules. Vitamin D acts as a modulator of this response by influencing intracellular pathways, notably the nuclear factor-kappa B (NF-κB) pathway.

The NF-κB is a central regulator of inflammation. In its inactive state, it is retained in the cytoplasm by an inhibitory protein, IκB. Upon stimulation, IκB is degraded, allowing NF-κB to translocate to the nucleus and initiate transcription of pro-inflammatory genes. Vitamin D may inhibit this process: VDR can directly interact with NF-κB proteins, reducing their transcriptional activity [58], and VDR deficiency is associated with increased NF-κB activity [59]. In the context of exercise, vitamin D3 supplementation has been shown to modulate the inflammatory response to high-intensity exercise-induced muscle damage, potentially via NF-κB inhibition [60].

**Figure 4.**
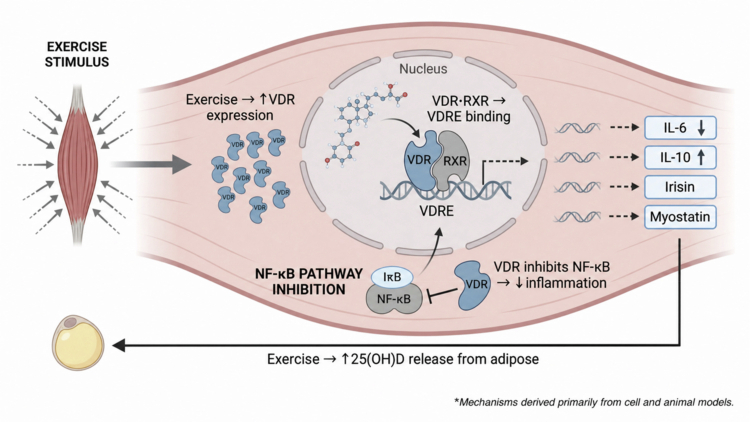
The Vitamin D-exerkine axis: proposed mechanistic crossroads. This figure illustrates the theoretical molecular mechanisms at the intersection of exercise and vitamin D signalling in skeletal muscle, derived primarily from in vitro and animal models. Exercise stimulus is shown to upregulates VDR expression. The active form of vitamin D (i.e. 1,25(OH)2D) binds the VDR, which forms a complex with RXR to potentially modulate the transcription of key exerkines (e.g. IL-6, IL-10, Irisin, Myostatin). Additionally, VDR activation is hypothesised to inhibit the pro-inflammatory NF-κB pathway.

By attenuating pro-inflammatory signalling through NF-κB, vitamin D may promote a more anti-inflammatory state. This dual suggests that vitamin D functions as a modulator, rather than a simple suppressor, of exercise-induced inflammation, helping to regulate responses necessary for adaptation while limiting excessive or prolonged inflammation. These findings support a role for vitamin D in fine-tuning cellular response to physical stress (Figure 4).

### 5.3. A bidirectional relationship: does exercise affect vitamin D levels?

The relationship between exercise and vitamin D extends beyond VDR regulation in muscle. Emerging evidence suggests that exercise may also influence systemic availability of vitamin D. Acute exercise has been associated with transient increase in circulating 25(OH)D. For example, Dzik et al. (2022) reported that a single session of intense exercise (e.g. Wingate Anaerobic Test) increased serum 25(OH)D3 concentration in trained adolescent boys shortly after exercise [61]. This observation has led to the hypothesis that exercise mobilises vitamin D from storage sites. As fat-soluble compound, vitamin D is stored in adipose tissue and, to a lesser extent, in skeletal muscle [15]. Exercise-induced lipolysis may facilitate the release of stored vitamin D into circulation [62]. Dzik et al. also observed concurrent increase in parathyroid hormone (PTH) and IL-6, suggesting coordinated hormonal and metabolic response to acute exercise [61]. Together, these findings suggest a dynamic, bidirectional interaction in which exercise may enhance both tissue responsiveness to vitamin D and its transient systemic availability.

## 6. Evidence synthesis: vitamin D's influence on key exerkines

This section synthesises current evidence on the regulatory influence of vitamin D on key exerkines. For each molecule, the discussion outlines its established role in exercise, summarises evidence of modulation by vitamin D, and describe proposed molecular mechanisms underlying these interactions. Table 2 provides a evidence map of vitamin D–exerkine interactions, summarising effect directionality, evidence tier (including athlete-specific data), putative mechanisms, and relevance for sports nutrition and athlete management. The table serves as a reference for the detailed discussions that follow.

### 6.1. Interleukin-6 (IL-6): the prototypical myokine

Role in Exercise: Interleukin-6 (IL-6) is a well-studied myokine with context-dependent effects. When released from contracting skeletal muscle during exercise, IL-6 primarily acts as a metabolic regulator, increasing glucose uptake and fatty acid oxidation in the muscle and enhancing hepatic glucose production [63]. This transient, exercise-induced increase is generally considered to have anti-inflammatory properties, in contrast to the chronically elevated, pro-inflammatory state of IL-6 observed in systemic inflammation.

Evidence for Vitamin D Modulation: Several studies have investigated the effect of vitamin D on the exercise-induced IL-6 response. A systematic review by Wyatt et al. [64] suggested that vitamin D supplementation may improve aerobic endurance in elite athletes, potentially related to immunomodulatory functions [64]. Another systematic review by Saedmocheshi et al. (2024) reported mixed findings, with some studies showing reductions in inflammatory markers and others reporting no significant change in IL-6 levels following supplementation [37]. Some studies further suggest that vitamin D supplementation may attenuate the post-exercise IL-6 responset [65].

Proposed Mechanism: Vitamin D is thought to regulate IL-6 partially through transcriptional mechanisms. The IL-6 gene promoter contains regions responsive to the VDR. Experimental evidence suggests that exercise-induced VDR expression in skeletal muscle may be associated with reduced IL-6 mRNA expression [57], indicating potential interference with transcriptional regulation following exercise.

### 6.2. Irisin: the “browning” myokine

Role in Exercise: Irisin, a myokine cleaved from the FNDC5 (Fibronectin type III domain-containing protein 5) precursor, has been studied for its role in metabolic regulation. Its primary described function is the “browning” of white adipose tissue, a process that is associated with increased energy expenditure and improved glucose homoeostasis [66]. Beyond its metabolic role, irisin has also been implicated in bone health, where it may promote osteoblast differentiation and bone formation.

Evidence for Vitamin D Modulation: A growing body of evidence suggests a positive association between vitamin D status and circulating irisin levels. However, because body composition and training environment may act as confounding factors, these observational findings do not establish a directional or causal relationship. Supporting this association, a study by Sanesi et al. (2023) reported that vitamin D supplementation increased serum irisin levels in patients with primary hyperparathyroidism [67]. The same study also showed that vitamin D treatment enhanced *FNDC5 expression* in myoblasts *in vitro*. Other studies have reported similar findings, suggesting that correcting vitamin D deficiency may increase in circulating irisin levels [68].

Proposed Mechanism: The molecular mechanism linking vitamin D to irisin production appears to be indirect may involve regulation of key metabolic pathways. The study by Sanesi et al. (2023) suggested that vitamin D-induced increase in *FNDC5* expression may be mediated through upregulation of Sirtuin 1 (Sirt1) and peroxisome proliferator-activated receptor-gamma coactivator 1-alpha (PGC-1α) [69]. PGC-1α is recognised as a key regulator of *FNDC5* expression. The proposed pathway is that vitamin D may increases Sirt1 expression, which in turn activates PGC-1α, leading to increased *FNDC5* transcription and subsequent production of irisin. While a direct binding site for VDR on the *FNDC5* promoter has not been clearly established, this indirect pathway provides a plausible mechanistic link.

### 6.3. Myostatin: the muscle growth gatekeeper

Role in Exercise: Myostatin (also known as GDF-8) is a member of the transforming growth factor-beta (TGF-*β*) superfamily and is recognised as a negative regulator of skeletal muscle mass. It inhibits myoblast proliferation and differentiation. Consequently, suppression of myostatin is considered a key factor in muscle hypertrophy in response to resistance exercise.

Evidence for Vitamin D Modulation: The relationship between vitamin D and myostatin appears to be inverse. Vitamin D deficiency has been associated with increased myostatin expression, while supplementation has been reported to reduce its expression. Koundourakis et al. (2016) reported that vitamin D suppresses myostatin expression, potentially contributing to its effects on muscle function [70]. More recently, Roizen et al. (2024) showed that high-dose vitamin D supplementation in mice was associated with reduced myostatin levels and a shift toward muscle growth over fat storage .

Proposed Mechanism: The suppressive effect of vitamin D on myostatin thought to involve transcriptional regulation. The promoter region of the myostatin gene contains regulatory elements responsive to transcription factors. It has been proposed that the activated VDR complex may interact with the myostatin promoter, potentially reducing its transcription. This suggests a possible molecular pathway through which vitamin D may influence muscle mass regulation.

### 6.4. Anti-inflammatory cytokines (IL-10): the immune balancer

Role in Exercise: Interleukin-10 (IL-10) is an anti-inflammatory cytokine that plays a role in resolving inflammation and maintaining immune homoeostasis. Following the initial pro-inflammatory response to exercise-induced muscle damage, IL-10 is released to attenuate the inflammatory cascade and promote a return to homoeostasis, which is important for tissue repair and adaptation.

Evidence for Vitamin D Modulation: Vitamin D is a recognized as a modulator of anti-inflammatory immune responses, and its effect on IL-10 has been studied in this context. Evidence suggests that vitamin D may increase IL-10 production by various immune cells, particularly regulatory T cells (Tregs). *In vitro* studies have shown that treatment with 1,25(OH)2D can promote differentiation of T cells into IL-10-producing Tregs [71]. In the context of exercise, Barker et al. [72] reported that individuals with sufficient vitamin D levels exhibited a greater post-exercise increase in IL-10 compared to those with insufficient levels [72].

**Figure 5.**
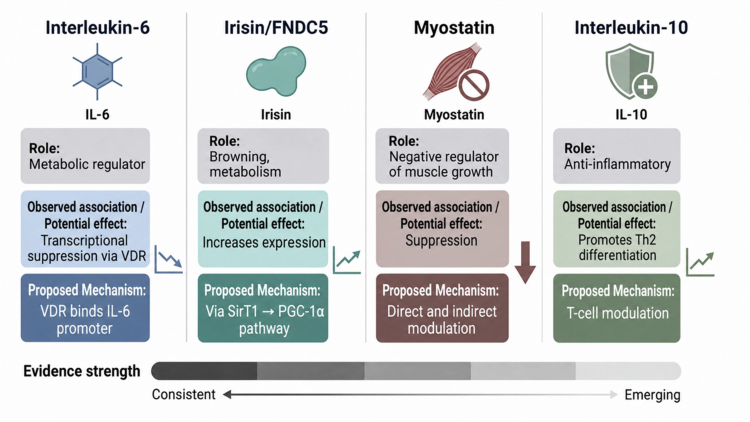
Evidence synthesis: potential influence of vitamin D on key exerkines. This figure provides a summary of the current evidence regarding the proposed modulatory effects vitamin D on four key exerkines. For each exerkine (IL-6, Irisin/FNDC5, Myostatin, and IL-10), it outlines its primary role, the observed associations with vitamin D status, the hypothesised mechanism of action, and a qualitative assessment of the current evidence strength (ranging from emerging to consistent), noting that much of the data is observational or derived from non-athlete populations.

Proposed Mechanism: The mechanism underlying vitamin D's effect on IL-10 production is thought to involve immune cell programming. VDR is expressed in immune cells, including T cells, and its activation may influence gene expression patterns that regulate cell differentiation and function. It is proposed that VDR activation promotes differentiation toward a regulatory T cell phenotype associated with IL-10 production [73]. This VDR-mediated effect may contribute to balancing the immune response by limiting excessive inflammation and promoting resolution (Figure 5).

**Table 2.** Vitamin D–exerkine evidence map relevant to athletes: directionality, evidence tier, putative mechanisms, and practical relevance.

Exerkine/mediator (primary source)	Direction with vitamin D status/supplementation	Evidence tier(s)* (athlete-specific?)	Putative mechanism(s) (summary)	Practical relevance for sports nutrition/athlete management	References
Interleukin-6 (IL-6) (skeletal muscle; immune)	↔ or ↓ post-exercise spike with supplementation in deficient individuals; no effect in sufficient individuals	Human RCT (athletes): mixed findings + mechanistic studies Athlete-specific: Yes (mixed)	VDR-mediated transcriptional regulation of IL-6 gene expression; vitamin D sufficiency associated with reduced IL-6 mRNA in muscle; modulation of NF-κB inflammatory pathway	Effect on post-exercise IL-6 response depends on baseline 25(OH)D status. Supplementation effects most apparent in deficient athletes (<20 ng/mL). Monitor baseline status; avoid causal claims regarding performance.	[27,37,58,61,66]
Irisin (FNDC5-derived) (skeletal muscle)	↑ association with vitamin D status; ↑ with supplementation in some populations	Human intervention + observational + cell culture Athlete-specific: Limited	Indirect pathway (demonstrated in vitro and human intervention): vitamin D → Sirt1/PGC-1α activation → ↑ FNDC5 transcription → ↑ irisin secretion	Metabolic adaptation signalling pathway plausible but requires validation in athlete populations. Hypothesis-generating for training adaptation research.	[29,67–69]
Myostatin (GDF-8) (skeletal muscle)	↓ or inverse association with vitamin D status	Animal supplementation + human observational Athlete-specific: Limited	Vitamin D supplementation associated with reduced myostatin signalling (animal models); mechanism in humans unclear	Preliminary evidence for muscle remodelling/hypertrophy signalling. Human athlete data needed; current evidence insufficient for practice recommendations.	[30,71]
Interleukin-10 (IL-10) (immune; exercise-responsive)	↑ anti-inflammatory response with vitamin D sufficiency post-exercise	Cell/immune + human exercise studies Athlete-specific: Some evidence	1,25(OH)₂D influences T-cell differentiation toward regulatory phenotypes; IL-10 production higher post-exercise in vitamin D-sufficient individuals	Immune resilience and inflammation resolution after intense training. Adequate vitamin D status may support recovery through anti-inflammatory signalling.	[36,72–74]
Vitamin D receptor (VDR) (skeletal muscle, immune cells)	↑ VDR expression with acute resistance exercise	Animal + human morphology studies Athlete-specific: Emerging	Exercise increases VDR protein in skeletal muscle; VDR expressed in muscle fibers mediates vitamin D signalling; supplementation may influence muscle VDR concentration	Bidirectional relationship: exercise increases VDR expression, potentially enhancing responsiveness to vitamin D supplementation. Timing of supplementation relative to training warrants investigation.	[45,46,56–58]
TNF-*α*/NF-κB–linked inflammation (immune)	↓ inflammatory signalling with vitamin D/VDR activity	Mechanistic + exercise-damage model Athlete-specific: Uncertain	VDR inhibits NF-κB activation by interacting with IκB kinase β; vitamin D3 modulates inflammatory response in exercise-induced muscle damage models	General anti-inflammatory mechanism with biological plausibility for exercise context. Clinical relevance to athlete recovery requires direct investigation.	[59–61]
Adiponectin/leptin (adipose-derived)	Adiponectin: ↑ with vitamin D Leptin: ↔ or ↓ in some contexts	Human metabolic studies (non-athletes) Athlete-specific: Uncertain	Adipokine regulation by vitamin D; associations confounded by adiposity in most studies	Body composition and metabolic context. Associations may be confounded by adiposity; independent effects in lean athletes unclear. Interpret cautiously.	[31–33]
FGF23–Klotho axis (bone–kidney endocrine)	Vitamin D regulates FGF23-Klotho axis (physiological feedback)	Human physiology studies Athlete-specific: Uncertain	Vitamin D regulates phosphate/vitamin D endocrine feedback loop via FGF23 and Klotho	Osteokine axis relevant to bone-kidney endocrine physiology. Potential relevance to bone adaptation and stress fracture risk in athletes requires investigation.	[35]

• Athlete-specific designations: - Yes (mixed): Multiple athlete studies with inconsistent findings. - Some evidence: 1-2 athlete studies available. - Limited: <2 athlete studies; preliminary data. - Emerging: Initial athlete data becoming available. - Uncertain: No direct athlete studies; extrapolated from non-athlete populations. **Abbreviations:** 1,25(OH)₂D, 1,25-dihydroxyvitamin D; 25(OH)D, 25-hydroxyvitamin D; FGF23, fibroblast growth factor 23; FNDC5, fibronectin type III domain-containing protein 5; GDF-8, growth differentiation factor 8; IL, interleukin; NF-κB, nuclear factor kappa B; PGC-1α, peroxisome proliferator-activated receptor gamma coactivator 1-alpha; RCT, randomised controlled trial; Sirt1, sirtuin 1; TNF-α, tumour necrosis factor alpha; VDR, vitamin D receptor.

^*^ **Evidence tier definitions:**• Cell/in vitro: Studies in cultured cells • Animal: Non-human animal models • Human observational: Association studies in humans • Human intervention/RCT: Supplementation or training trials in humans.

### 6.5. Other emerging players (BDNF, FGF21, SPARC)

Beyond the well-established exerkines, preliminary evidence suggests that vitamin D may influence a broader range of exercise-induced signalling molecules, representing a emerging area in exerkine research.

Brain-Derived Neurotrophic Factor (BDNF): BDNF is a neurotrophin upregulated by exercise and involved in neuronal health and cognitive function. Some studies suggested a potential synergistic effect of exercise and vitamin D on BDNF levels, although the underlying mechanisms, particularly in muscle-derived BDNF, remain unclear [74].

Fibroblast Growth Factor 21 (FGF21): FGF21 is a metabolic regulator involved in glucose and lipid metabolism. Although its interaction with vitamin D in the context of exercise is not well defined, both are associated with metabolic regulation, suggesting a possible crosstalk.

Secreted Protein Acidic and Rich in Cysteine (SPARC): SPARC is an exercise-induced protein implicated in muscle regeneration and function. While interaction between vitamin D and SPARC have been reported in other contexts, such as cancer, their role in exercise-related physiology remain to be determined.

Overall, these emerging links suggest a broad and incompletely understood landscape of the vitamin D–exerkine axis. Further research is required to clarify the underlying mechanisms and physiological relevance of these interactions.

## 7. Clinical implications and the integrated athlete

The mechanistic links between vitamin D and the exerkine network have direct clinical relevance for athletes. Beyond its role in bone health, vitamin D status may act as a modulator of the body's response to training, influencing adaptation, recovery, immune function, and injury risk. This section integrates the preceding evidence into a framework for athlete populations. Table 3 provides practitioner-oriented guidance for vitamin D assessment, supplementation, and monitoring, translating current evidence into practical approaches for testing, dosing, and safety.

**Table 3.** Practitioner guidance for vitamin D in athletes: testing, timing, targets, dosing, and safety.

Guidance question	Clinical approach	Operational details	References
Who to test?	Prioritise high-risk athlete groups	Athletes at elevated risk: • Indoor training environments • Winter months at latitudes > 35°N or S • Darker skin pigmentation (Fitzpatrick types IV-VI) • History of stress fractures • Frequent upper respiratory infections (>3 episodes/year) Consider baseline screening for all athletes at start of training season.	[13,15,16,53–55]
When to test?	Use seasonal timing + pre-season screening	For outdoor athletes in temperate climates: • Test twice yearly: late winter/early spring (nadir) and late summer/early autumn (peak) For indoor athletes or year-round supplementation: • Test at pre-season and mid-season Note: Seasonal pattern applies to athletes with regular outdoor sun exposure; indoor athletes may not exhibit seasonal variation.	[54,75,76]
What to measure?	Measure serum 25(OH)D	Report in both ng/mL and nmol/L (conversion: 1 ng/mL = 2.5 nmol/L). Interpret with context: • Season (expect 10-20% lower in winter) • Recent sun exposure • Current supplementation status • Training environment (indoor vs. outdoor)	[75,76]
Target levels	Maintain minimum; consider higher “preferred” range	Minimum for skeletal health: ≥ 30 ng/mL (75 nmol/L) [IOM] Higher targets proposed for immune function and muscle performance: 40-50 ng/mL (100-125 nmol/L) [based on observational data; RCT evidence in athletes limited] Individualise based on: • Sport type and training phase • Individual response to supplementation • Clinical outcomes (illness frequency, injury rate)	[52,77,78]
Correction dosing	Short-term higher dosing with re-test	For serum 25(OH)D < 20 ng/mL (50 nmol/L): • 5,000 IU/day vitamin D3 for 8-12 weeks For serum 25(OH)D 20-30 ng/mL (50-75 nmol/L): • 3,000 IU/day vitamin D3 for 8-12 weeks Re-test after correction period; target ≥ 30 ng/mL (75 nmol/L). If levels remain < 30 ng/mL after 12 weeks, consider higher doses (up to 10,000 IU/day) under medical supervision or investigate malabsorption.	[78–80]
Maintenance dosing	Individualised maintenance	1,000-2,000 IU/day: • Athletes with regular outdoor training in sunny climates 2,000-4,000 IU/day: • Indoor athletes or high latitudes • Increase by 1,000 IU/day during winter months if seasonal decline observed Individualise based on re-testing every 3-6 months; adjust dose to maintain target range.	[75,76]
Safety/upper limits	Avoid indiscriminate high-dose use; monitor toxicity risk	Tolerable upper intake level (UL): 4,000 IU/day for prolonged use [IOM/NASEM] Short-term higher doses (up to 10,000 IU/day) may be used for deficiency correction under medical supervision [Endocrine Society guideline] Monitor for hypercalcemia if using doses > 4,000 IU/day for extended periods. Toxicity rare below 10,000 IU/day in healthy individuals.	[80]
Implementation	Integrate within holistic athlete plan	Comprehensive approach: • Integrate vitamin D assessment into nutrition screening • Encourage 10-30 minutes midday sun exposure 2-3 × /week when feasible (balance skin cancer risk) • Coordinate supplementation with training periodisation • Consider higher doses during high-volume training or elevated illness risk Educate athletes: Vitamin D supplementation effects are permissive (enabling optimal function) rather than ergogenic (directly enhancing performance) in the absence of deficiency.	[75,76]

**Note:** Guidance based on current evidence and expert consensus; individual athletes may require modified approaches based on baseline status, training demands, and clinical response. Medical supervision recommended for athletes with conditions affecting vitamin D metabolism (e.g. malabsorption, chronic kidney disease). Dosing recommendations are based on skeletal health targets, not exerkine optimisation. **Abbreviations** 25(OH)D, 25-hydroxyvitamin D; D3, cholecalciferol; IOM, Institute of Medicine; IU, international units; NASEM, National Academies of Sciences, Engineering, and Medicine; RCT, randomised controlled trial; UL, tolerable upper intake level.

### 7.1. Training adaptation and recovery

Effective training is a balance between stimulus and adaptation. Evidence suggests that sufficient vitamin D status may support training adaptations by influencing the molecular condition of skeletal muscle.

The potential modulation of myostatin and irisin represents a theoretical pathway for this process. In animal model, by suppressing myostatin, a negative regulator of muscle mass, vitamin D has facilitated greater hypertrophic response to resistance training [70]. Concurrently, increased irisin levels may promote a more favourable metabolic profile, including improved glucose homoeostasis and energy expenditure, which support sustained training capacity [69]. Together, these changes may contribute to an anabolic and metabolically efficient condition conducive to positive training adaptations.

Recovery from strenuous exercise is equally important. The inflammatory response to exercise-induced muscle damage must be tightly regulated, as an excessive or prolonged inflammation may impair recovery and increase the risk of overtraining. Vitamin D may contribute to this regulation by modulating pro- and anti-inflammatory cytokine response. Specifically, it has been associated with attenuation of post-exercise IL-6 and increased IL-10production [72,81]. The balanced response may support muscle repair, reduced delayed onset muscle soreness (DOMS), and facilitate a faster return to training.

### 7.2. Immune resilience and reduced illness risk

Athletes, particularly those engaged in heavy training, often experience transient immune suppression, increasing susceptibility to infections such as upper respiratory tract infections (URTIs). Evidence suggests that vitamin D may play a role in mitigating this risk through both direct immune effects and indirect modulation of exerkines.

Vitamin D has documented effects on the immune function, including enhancement of immune system and modulation of T-cell activity [13]. These effects may contribute to improved host defence. In combination with its influence on the exerkine profile, vitamin D may help regulate inflammatory balance. Specifically, attenuation of pro-inflammatory cytokines such as IL-6, alongside increased anti-inflammatory cytokines such as IL-10, may promote a more controlled immune response [82]. Maintaining this balance may reduce excessive inflammation and potentially contribute to lower incidence, severity, or duration of URTIs in athletes, thereby minimising disruptions to training.

### 7.3. Injury prevention

The prevention of bone and muscle injuries, is a primary concern in any athlete. Vitamin D, through its established role in musculoskeletal health and potential modulation of the exerkine axis, may contribute to injury prevention.

Stress fractures, a common overuse injury, results from an imbalance between bone microdamage and repair. Vitamin D plays a central role in in calcium homoeostasis and bone mineralisation, which are essential for maintaining bone integrity [83]. In addition, its association with exerkines such as irisin which has been linked to bone formation, suggest a potential role in supporting skeletal resilience [69].

Muscle injuries are also prevalent in athletes. Vitamin D has multiple role muscle function and adaptation. By suppressing myostatin, it may contribute to muscle strength and structural integrity [70]. Furthermore, by modulating the inflammatory response to exercise, vitamin D may help limit muscle damage and support repair process, potentially reducing the risk of strains or tears [81].

**Figure 6.**
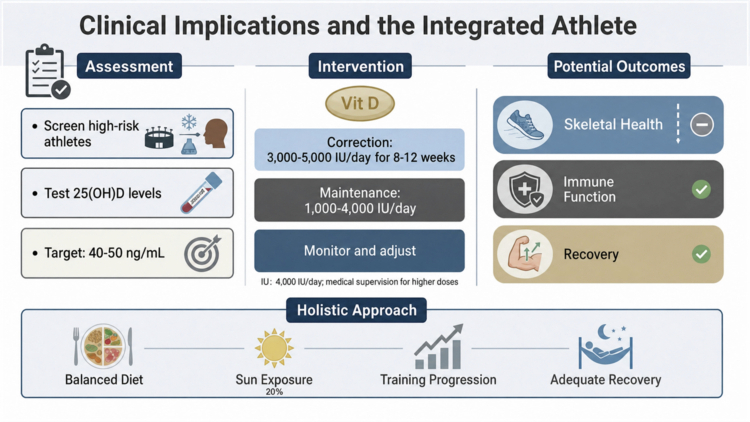
Clinical implications and the integrated athlete. This figure presents a clinical framework for managing vitamin D status in athletes. It is divided into three sections: (1) Assessment: Screening high-risk athletes and testing 25(OH)D levels to a target of 40-50 ng/mL (2) Intervention: Recommended supplementation protocols for correction and maintenance. (3) Outcomes: The theoretically supported benefits for immune function, skeletal health, and recovery. The bottom banner emphasises a holistic approach that integrates diet, safe sun exposure, training, and recovery. Note that while optimising vitamin D is foundational for athlete health, direct ergogenic effects on performance via exerkine modulation remain unconfirmed.

Overall, the combined effects of vitamin D on bone and muscle, through both direct mechanisms and interaction with exerkine network, suggest potential role in reducing overall injury risk in athletes (Figure 6).

## 8. Practical recommendations for practitioners

The translation of evidence on the vitamin D-exerkine axis into actionable guidance is essential for bridging laboratory findings and athletic performance. This section provides practical recommendations for practitioners working with athletes, including vitamin D assessment, supplementation strategies, and integration into a comprehensive athlete care model.

### 8.1. Testing and status assessment

Who to Test: Given the high prevalence of vitamin D insufficiency in athletes, a targeted testing strategy is warranted. Screening should be prioritised for high risk group, including athletes train primarily indoors (e.g. basketball players, swimmers, gymnasts), those training during winter at higher latitudes, and athletes with darker skin pigmentation, which reduces cutaneous vitamin D synthesis [84]. Athletes with a history of stress fractures or recurrent illnesses should also be considered for testing.

When to Test: Timing is an important consideration for effective management. Pre-season screening allows identification and correction of deficiencies before competitive period. For at-risk athletes, testing twice a year is commonly recommended: once in the late winter or early spring to capture the seasonal nadir, and again in the late summer or early autumn to assess peak levels [85]. This approach supports more individualised supplementation strategies.

Target Levels: Although the optimal serum 25-hydroxyvitamin D [25(OH)D] concentration for athletes remain debated, evidence suggests levels should exceed general population threshold. A minimum level of 30 ng/mL (75 nmol/L) is widely considered appropriate, with some experts suggesting a target range of 40-50 ng/mL (100-125 nmol/L) to support potential non-skeletal benefits [75,79].

### 8.2. Supplementation strategies

Dosing: Supplementation strategies should be individualised based on baseline 25(OH)D levels. For deficiency (<20 ng/mL) or insufficiency (20-30 ng/mL), higher initial doses are often required. A common approach is 3,000-5,000 IU/day of vitamin D3 for 8-12 weeks, followed by re-testing to assess whether target levels have been achieved [77]. Once sufficiency is reached, a maintenance dose of 1,000-4,000 IU/day is often adequate, although adjustments may be needed based on season, training environment, and individual factors [78].

Safety: Vitamin D supplementation is generally safe but require appropriate monitoring. The tolerable upper intake level (UL) is set at 4,000 IU/day by the Institute of Medicine, while the Endocrine Society suggests that up to 10,000 IU/day is safe for short-term use in deficient individuals under medical supervision [76]. Excessive intake should be avoided. as toxicity, though rare, can lead to hypercalcemia. Supplementation, particularly at higher doses, should therefore be guided by serum 25(OH)D measurements and supervised by a qualified healthcare practitioner.

### 8.3. A holistic approach

Vitamin D optimisation should be considered within a broader context of athlete care. Supplementation is not a substitute for a balanced diet, appropriate training progression, and adequate recovery. While correcting deficiency may remove a barrier to optimal performance, it is not a standalone solution. A holistic approach, including a nutrient-dense diet with natural vitamin D sources (e.g. fatty fish, fortified dairy), appropriate sun exposure, and well-structured training and recovery practices, remains the cornerstone of athlete health and performance.

## 9. Research gaps and future directions

The vitamin D-exerkine axis represents an evolving area of study. While current evidence suggests a biologically plausible connection, important gaps remain. The following sections outline key research gaps and future directions to further clarify this relationship.

### 9.1. Unexplored exerkines

Current research has predominantly focused on a limited number of well-characterised myokines, such as IL-6, irisin, and myostatin. However, the exerkine family is extensive and includes molecules secreted from various tissues, including hepatokines, adipokines, and osteokines. The extent to which vitamin D modulates these broader exerkine classes remains unclear [80,86].

Future studies should expand the analytical scope to include a wider range of exerkines. Investigating the effects of vitamin D on hepatokines involved in glucose metabolism and adipokines regulating appetite and inflammation may provide a more comprehensive understanding of its systemic role in exercise physiology.

### 9.2. Dose-response relationships

While this review suggests that vitamin D status may influence exerkine profiles, the dose-response relationship remains unclear. It is uncertain whether this relationship is linear or characterised by a threshold effect beyond which additional increases in vitamin D confer limited benefit. It is also possible that different exerkines have different optimal vitamin D concentrations.

Future research should incorporate multiple supplementation dosages to better define these relationships. Such work may help refine target 25(OH)D levels for athletes, moving beyond a general sufficiency toward target optimised for specific physiological outcomes [87,88].

### 9.3. Sex-specific and sport-specific differences

Most research in sports science, including studies on vitamin D and exerkines, has been conducted in male athletes. There is a clear lack of data on whether these interactions differ between male and female athletes. Given the known differences in endocrine function, body composition, and metabolism, the vitamin D-exerkine axis may also exhibit sex-specific characteristics [54].

Similarly, different sport types (e.g. endurance vs. strength/power) elicit distinct exerkine responses. Future studies should directly compare these groups to determine whether vitamin D's effects differ by sex or sport. Such work may support more the development of more personalised nutritional strategies.

### 9.4. Intervention studies

Much of current evidence linking vitamin D and exerkines is correlational, highlighting the need for well-designed randomised controlled trials (RCTs). Future studies should combine controlled exercise interventions with vitamin D supplementation and assess a broad panel of exerkines as primary outcomes [89]. Such approaches may help clarify causal relationship and underlying mechanisms. Furthermore, incorporating tissue-level analyses (e.g. muscle biopsies) may provide insight into the effects of vitamin D on VDR expression and exerkine genes transcription within target tissues.

### 9.5. Timing and chrononutrition

The emerging field of chrononutrition, which examine the timing of nutrient intake in relation to circadian rhythms, offers a potential avenue for research. It remain unclear whether the timing of vitamin D supplementation relative to exercise influences exerkine response. For example, pre-vs post-exercise supplementation or time-of-day effects may yield different outcome.

Addressing these questions will require well-controlled studies integrating both exercise and nutrient timing [90]. Investigating these chronobiological aspects may help refine vitamin D supplementation strategies in athletic populations.

## 10. Conclusion

This review synthesised current molecular and clinical evidence on the relationship between vitamin D and the exercise-induced secretome. Overall, the findings suggest that vitamin D may act as a systemic modulator of signalling pathway involved in the response to physical training, extending beyond its established role in bone health. However, direct causal evidence in athletic population remains limited.

### 10.1. Summary of the vitamin D-exerkine axis

Evidence from cell and animal model suggests that vitamin D, through its nuclear receptor (VDR), may influence the expression of selected exerkines. VDR activation has been linked to reduced expression of the pro-inflammatory cytokine IL-6 and increased production of the anti-inflammatory cytokine IL-10. Vitamin D has also been associated with increased expression of irisin and reduced myostatin, a negative regulator of muscle growth. In parallel, exercise influence circulating vitamin D levels, suggesting a potential feedback interaction within exercise-response system.

In human studies, observational data indicate associations between vitamin D status and circulating exerkine levels. A bidirectional relationship is plausible: exercise may increase vitamin D availability through mobilisation from storage tissues, while vitamin D sufficiency may influence inflammatory and metabolic responses to exercise. However, these associations do not establish causation, and intervention studies examining exerkine modulation in athletes remain limited.

Overall, the proposed intersection between vitamin D and exerkine pathways offer a novel, hypothesis-generating framework for future research. However, current evidence does not support the premise that vitamin D supplementation enhances athletic performance, recovery or training adaptation through the modulation of these mediators.

### 10.2. A new perspective for sports nutrition—with important caveats

A central limitation of this review is the nature of the underlying evidence base. The mechanistic foundation of the proposed vitamin D–exerkine axis rests primarily on cell and animal studies, which allow controlled investigation of VDR signalling but cannot be directly extrapolated to trained human athletes. Human evidence is largely observational and derived from non-athlete populations, including elderly adults, patients with metabolic disease, and recreationally active individuals. Athlete-focused intervention trials assessing exerkines as primary outcomes remain limited. The focus on athletic populations therefore reflects the translational intent of the proposed axis rather than the current strength of athlete-specific causal evidence. Practical recommendations should be interpreted with this limitation in mind.

Recognition of potential vitamin D–exerkine interactions prompts reconsideration of current sports nutrition frameworks. The conventional view of vitamin D as primarily involved in calcium metabolism and bone health may be incomplete. Available evidence suggests that optimising vitamin D status could be considered within a broader strategy to support physiological response to training.

However, several limitation should be acknowledged. First, most mechanistic evidence derives from cell culture and animal models, and extrapolation to human athletes remains uncertain. Second, most human studies have been conducted in non-athlete populations (e.g. elderly, obese, metabolic disease patients) or have not measured exerkine outcomes. Athlete-focused trials are limited and have primarily examined performance or immune function, with inconsistent findings. The extent to which vitamin D modulates exerkines in trained athletes, and whether this has functional relevance, remains uncertain.

Third, observational associations between vitamin D status and exerkine levels are subject to confounding including difference in sun exposure, training environment, body composition, and diet. Establishing causality requires well-controlled randomised trials. Fourth, optimal vitamin D levels for exerkine modulation are not defined and may differ from threshold established for skeletal health.

Given these limitations, vitamin D optimisation should be viewed as a foundational element of athlete health, supporting skeletal integrity and immune function, rather than a direct ergogenic strategy. While sufficient vitamin D may help remove a potential barrier to optimal adaptation, current evidence does not support claims that supplementation enhance performance via exerkine modulation.

Overall, vitamin D may be considered a candidate modulator of exercise-responsive signalling, but its practical relevance requires confirmation in well-designed studies involving in athletic populations.

### 10.3. Future directions: from plausibility to evidence

Three key evidence gaps warrant attention. First, no published randomised controlled trial has assessed exerkine concentrations as primary outcomes in response to vitamin D supplementation in trained athletes, representing a major methodological gap. Second, dose–response data are largely absent; it remains unclear whether relationships between serum 25(OH)D levels and exerkine modulation are linear, threshold-based, or exerkine-specific, and what concentrations are optimal for exercise-related outcomes. Third, sex-specific analyses are limited. Given known differences in vitamin D metabolism, VDR expression, and exerkine profiles, sex-stratified studies are needed before generalisable recommendations can be made.

The vitamin D–exerkine axis, outlined in this review, represents a hypothesis-generating framework rather than an established paradigm. Advancing from biological plausibility to evidence-based application requires several research priorities:

- Athlete-specific randomised controlled integrating exercise interventions with vitamin D supplementation, with comprehensive exerkine profiles and control for baseline vitamin D status.
- Mechanistic human studies using muscle biopsies to assess VDR expression, exerkine gene transcription, and protein secretion in response to supplementation.
- Dose-response studies to determine whether relationships are linear, threshold-based, or exerkine-specific, and to define optimal 25(OH)D targets for athletic populations.
- Sex-specific and sport-specific investigations to evaluate potential difference between male and female athletes across sport types.
- Longitudinal studies examining association between vitamin D status, exerkine profiles and outcomes such as training adaptation, recovery, illness, and injury over time.

### 10.4. Final thought

The interaction between nutritional status and physical activity is a key modulators of human physiology. The potential relationship between vitamin D and the exerkine network represents one example of this interaction. Optimising nutritional status with structured training may support physiological function, health, and performance.

However, translating this concept into practice requires further investigation. The vitamin D-exerkine axis remains a hypothesis-generating framework, and its relevance to athletes has not been established through rigorous studies. Until such evidence is available, vitamin D optimisation should be guided by its established role in skeletal health and immune function, while potential effects via exerkine modulation remain unconfirmed.
